# Minimizing sample bias due to stigmatized behaviours: the representativeness of participants in a cohort study of alcohol in pregnancy

**DOI:** 10.1186/s12874-022-01629-2

**Published:** 2022-05-13

**Authors:** David Tappin, Daniel Mackay, Lucy Reynolds, Niamh Fitzgerald

**Affiliations:** 1 Section of Child Health, College of Medical, Veterinary & Life Sciences, Wolfson Medical School Building, University of Glasgow, 31, Shawhill Road, Glasgow, Scotland G41 3RW UK; 2 Institute of Health and Wellbeing, Public Health, University of Glasgow, 1 Lilybank Gardens, Glasgow, Scotland G12 8RZ UK; 3 NHS Greater Glasgow & Clyde Health Board, Community Child Health, Children’s Services, Woodside Health and Care Centre, 891 Garscube Road, Glasgow, G20 7ER UK; 4 Institute for Social Marketing & Health, Faculty of Health Sciences & Sport, University of Stirling, Stirling, FK9 4LA UK; 5 SPECTRUM Consortium, Edinburgh, UK

**Keywords:** Ethics of informed consent, Clinical markers, Cohort study, Data collection, Demography, Epidemiology

## Abstract

**Background:**

Stigmatized behaviours are often underreported, especially in pregnancy, making them challenging to address. The Alcohol and Child Development Study (ACDS) seeks to inform prevention of foetal alcohol harm, linking self-report as well as a maternal blood alcohol biomarker with child developmental outcomes. Samples were requested using passive, generic consent. The success of this approach at minimizing bias is presented comparing characteristics of women who provided samples to those who did not.

**Methods:**

All pregnant women in the study city were sent a Patient Information Sheet (PIS) with their first NHS obstetric appointment letter. The PIS informed them that the NHS would like to take an extra blood sample for research purposes, unless they opted out. Neither the women nor the midwives were informed that the samples might be tested for an alcohol biomarker. This paper examines the extent to which women who provided the extra sample were representative of women where no sample was provided, in terms of routinely collected information: age; body mass index; area-based deprivation; previous pregnancies, abortions and caesarians; smoking status and carbon monoxide level; self-reported alcohol use, gestation and birth weight of their baby. Chi-square and Mann-Whitney U tests were used to compare groups.

**Results:**

3436 (85%) of the 4049 pregnant women who attended their appointment provided the extra sample. Women who did not were significantly younger (*p* < 0.001), more materially deprived (*p* < 0.001), and less likely to be considered for intervention based on self-reported alcohol use (*p* < 0.001). There were no significant differences between the two groups on other routine data.

**Conclusions:**

The use of passive consent without disclosure of the specific research focus resulted in a high level of sample provision. There was no evidence that study blinding was breached, and women who provided a sample were more likely to report alcohol consumption. Passive consent to draw additional blood for research purposes at routine antenatal venipuncture reduced sampling bias compared to asking women to give blood for an alcohol study. This methodology may be useful for other stigmatised behaviours.

## Background

Drinking alcohol in pregnancy can cause a wide range of neurodevelopmental and other harms to the embryo/foetus and is a leading, but preventable, cause of birth defects, with lifelong implications [1]. Globally, the estimated prevalence of Fetal Alcohol Spectrum Disorders (FASD) is 7.7 per 1000 in the general population, with the highest prevalence in the European Region at 19.8 per 1000 [2]. FASD is associated with a wide range of comorbidities [3], resulting in high costs for both children and adults [4]. Appropriate prevention responses are therefore a public health priority. The World Health Organization recommends that health professionals ask all women about their alcohol use when they attend antenatal care [5], however many women under-report their consumption [6] and it can be difficult for midwives to identify those at risk [7]. A recent UK study which tested samples of meconium for alcohol biomarkers found at least a five-times higher prevalence of in-pregnancy drinking than with self-report data from the same women [8]. Improved screening methods are needed to identity those at risk and maximise maternal and child health.

A recognized challenge to research on alcohol consumption in pregnancy, is that women who consent to take part in studies may not be representative of the wider population. Drinking alcohol in pregnancy is highly stigmatized in the UK and many other cultures, and pregnant women who are drinking may feel ashamed or defensive [9]. Furthermore, women report a fear of judgmental attitudes [10] or repercussions for them as parents including intervention by child protection services [11]. Women drinking in pregnancy may therefore be least likely to take part in a study of this topic. This problem has hampered the development of accurate screening methods for drinking in pregnancy, as it has not been possible to identify a gold standard or ‘true positive’ measure of alcohol consumption in pregnancy – against which any new methods could be compared or which could be used to study the effectiveness of interventions to prevent or reduce such consumption [12, 13]. Other studies of alcohol consumption have faced similar challenges, whereby members of the group of interest are more likely to decline to take part in the study, biasing the sample [14].

The aim of the Alcohol and Child Development Study (ACDS) was to use routinely collected data supplemented by a recognized biochemical marker of heavy alcohol use to predict poor developmental outcomes in offspring. This paper examines whether passive consent to draw additional blood for research purposes reduces sampling bias compared to past studies that directly asked for a blood sample for an alcohol study.

## Methods

The Alcohol and Child Development Study (ACDS) involves a cohort of pregnant women in a UK city and aims to inform the development of improved methods of identification of women at risk of having a child affected by foetal alcohol exposure. The study will anonymously follow the children of a cohort of women identified in pregnancy, to examine whether or not there is a relationship between either positive maternal blood tests for alcohol biomarkers and/or self-reported alcohol consumption in pregnancy, and later childhood developmental disorders. If women drinking in pregnancy opted out of the cohort, such as for the reasons described above, the ACDS would be unable to achieve its aims.

### Design

This representativeness study compares routinely collected data from pregnant women attending ‘booking’ appointments for maternity care in the study city, who had an extra blood sample taken for the ACDS, with those who did not.

### Setting

The setting for the ACDS was midwife-led first maternity clinic appointments at 13/14 weeks of pregnancy in a medium sized UK city between June 2017 and June 2018 (‘the study period’). These appointments are to start the midwife-led pregnancy support programme and include a formal pregnancy test, breath test for smoking as well as routine blood tests for infection with hepatitis B, syphilis and HIV for which current treatment improves birth outcomes. Women are also asked about alcohol use and provided with a brief intervention or referral to specialist services to support alcohol reduction and elimination during pregnancy. The data analysed in this study is from all the maternity booking clinics where the extra sample collection was a routine part of the maternity booking appointment.

### Participants and information provided

All pregnant women reporting to the NHS in the study city during the study period received a study information sheet along with the letter informing them of their booking appointment date and time and all women who attended during the analysis period are included in the analysis for this paper as outlined above. In line with the ethical approval conditions, the information sheet (available on request) informed women that the NHS wished “*to collect an extra 2ml (one third of a teaspoon) of blood from all pregnant women”,* and that this extra blood would be “*used anonymously for research … which may help us to learn more about the best ways to support the health of women and babies and improve our service.*” Women were informed that they could opt out of the study by letting their midwife know that they did not want the sample taken. A contact name and number was provided on the PIS for any queries.

Similar generic information was provided to all midwives by a senior research midwife unconnected to the study team, and they were advised on when and how to take the additional sample. Midwives were asked not to actively consent the women before taking the extra sample, but to take the sample ‘routinely’ unless the woman voiced her wish to opt out. The potential testing of the additional blood sample for an alcohol biomarker was not disclosed to women or frontline midwives.

The blood samples were stored in a sample repository with a study identifier linked to routine maternity booking data held by a secure NHS data service such that neither the researchers nor clinicians could identify who had a sample taken. The study aimed to assay these samples for a marker used by the Driver and Vehicle Licensing Agency (https://www.drinkdriving.org/cdt-alcohol-test.php) to assess current heavy alcohol intake– Carbohydrate Deficient Transferrin [15].

### Quantitative variables and data sources

Detailed self-report of alcohol use was collected at the first maternity visit via a locally developed computerised data system until 31st October 2017. A change in computer system from November 2017 meant that less detailed self-reported data was recorded regarding alcohol use during pregnancy. For this reason, this paper analyses data only from appointments during the period 12th June 2017 and 31st October 2017 (‘the analysis period’) to enable comparison of self-reported alcohol consumption.

With the exception of birth weight of the baby, variables were gathered from routinely collected maternity service data recorded directly by midwives onto a computerised database. Only those variables which were deemed not to risk the anonymity of participants was shared with researchers. Shared data included the following:

- Area-based material deprivation, [16] calculated from patient postcode using government statistics.
- Age
- Height and weight at the time of booking, used to calculate Body Mass Index (BMI) using the formula Weight (in kilos)/Height^2^ (in metres).
- Number of previous pregnancies, previous spontaneous abortions, previous therapeutic abortions.
- Smoking history, self-reported smoking while pregnant, carbon monoxide breath test level
- Estimated gestation in weeks calculated from recall of last menstrual period, and
- Self-reported alcohol use. This was recorded in 16 domains, of which just four of these contained sufficient data for the safe data facility to be sure that anonymity would be retained. How much did you drink daily before pregnancy? (converted into alcohol units); Brief Intervention was required Yes/No?; Referral to (alcohol) intervention nurse Yes/No?; How much do you drink each week now? (converted into alcohol units).
- The birth weight of the baby was obtained through record linkage with a national database.

All of this data was transferred to and held by an NHS safe data facility to enable future linkage with child health records without compromising the anonymity of the participants or their children.

### Bias

The unusual consent procedures in this study described above – passive consent for generic research using a routine extra blood sample - were designed to minimise sources of bias in the cohort. This paper compares those who provided an extra sample with those who did not provide an extra sample to assess potential bias.

### Study size

The whole cohort from 12th June 2017 to 30th June 2018 was chosen to include all festivals and holiday periods during a full calendar year when alcohol intake may increase.

For this analysis only women who booked during the months of June, July, August, September and October 2017 were included. After this period the computerized data collection programme was replaced by another system with less detail regarding self-report of alcohol use. The representativeness being assessed in this first 4.5 months regarding alcohol use is likely to pertain to the whole 12 month cohort as nothing else changed apart from the self-report data collected.

### Statistical methods

Statistical analyses were performed with Stata 12.10 [17]. Descriptive statistics (percentages, medians and inter-quartile ranges) were derived for all variables. Univariate statistical comparison used Pearson X^2^ test for linear trend and Mann-Whitney U tests to ascertain possible group differences between women who had an extra sample collected and those who did not. Logistic regression was used to determine whether alcohol intake predicted non-delivery of a blood specimen after controlling for age category and deprivation quintile.

### Ethics

Ethics approval was granted by the local NHS Research Ethics Committee (approval number provided, but not published to protect the identity of the study site). The study team attended the committee meeting at which the study was considered in person, and discussed it with the committee. The case made for approval was that: (1) the study was very unlikely to be worthwhile if full informed consent was required for participation; (2) the analysis in the study would be entirely anonymous; (3) the extra sample would be taken at the same time as other routine blood samples, thus not requiring additional venipuncture; (4) the risks of participation were therefore minimal; and critically (5) there could be significant potential benefits in terms of better prevention of a common and serious childhood developmental disorder, should the study be successful. The researchers successfully argued that the potential benefits of the study at population level outweighed any harm caused by providing individual women with incomplete information about the study. It was decided that frontline midwives would also not be informed about the true purpose of the sample, so that they would not be able to reveal the true purpose even if asked (and there would be no question of them having to choose between revealing or concealing it from women).

Furthermore, to reduce the burden on midwives, and to ensure an adequate sample size, the ethics committee agreed to passive consent, whereby women were informed about the study in generic terms via a Patient Information Leaflet, PIS, but that the extra blood would be taken unless women actively voiced their wish for it not to be. Formal written consent was therefore not required. Women could proactively opt-out of the study based on reading the PIS, but the midwife was not instructed to ask for explicit permission to take the extra blood for this study. The sponsor for the study was the local NHS management authority.

## Results

To our knowledge, no one called the telephone enquiries number on the PIS with any queries about the study, and no difficulties were reported to local research midwives regarding the collection of the sample by frontline midwives.

During the analysis period (12th June 2017 to 31st October 2017), 3436 (85%) of the 4049 women who attended the included maternity clinics for their antenatal booking appointment had the extra ‘research’ blood sample collected.

As shown in Table 1, the 15% of women who did not have an extra research blood sample collected were significantly younger (*p* < 0.001), more materially deprived (*p* < 0.001), and less likely to require support for self-reported alcohol use during pregnancy (*p* < 0.001) than women from whom a sample was collected. Running a multivariable logistic regression of alcohol use in pregnancy whilst controlling for age category and deprivation quintile, showed that women who disclosed alcohol use in pregnancy were 37% more likely to provide a blood sample (OR = 1.37, 95% CI (1.12, 1.68), *P* = 0.002) than those who did not disclose alcohol use.

**Table 1:** Comparison of women who had an extra research blood sample collected at routine venipuncture with those who did not in terms of routine collected data

	Did not have an extra sample taken (613)	Had an extra sample taken (3583)	***P*** Value
**Area based deprivation, SIMD quintile, 1 = most deprived; 5 = least deprived**			
SIMD 1	266 (46.2)	1306 (38.0)	
SIMD 2	105 (18.2)	604 (17.6)	
SIMD 3	73 (12.7)	435 (12.7)	
SIMD 4	64 (11.1)	484 (14.1)	
SIMD 5	68 (11.8)	607 (17.7)	
Missing	37	147	< 0.001
**Age category (years)**			
15 to 20 years	30 (5.1)	128 (3.6)	
20 to 30 years	234 (39.5)	1406 (39.5)	
30 to 40 years	306 (51.6)	1860 (52.3)	
40 to 50 years	23 (3.9)	165 (4.6)	
Missing	20	24	< 0.001
**BMI (kg/m sq)**			
Underweight	15 (4.4)	57 (3.1)	
Normal	160 (47.3)	901 (48.8)	
Over weight	163 (48.2)	889 (48.1)	
Missing	275	1736	0.704
**Previous pregnancies**			
0	231 (51.9)	1289 (54.5)	
1	113 (25.4)	607 (25.6)	
2	52 (11.7)	250 (10.6)	
3	20 (4.5)	112 (4.7)	
4+	29 (6.5)	109 (4.6)	
Missing	168	1216	0.121
**Previous spontaneous abortions**			
0	350 (78.5)	1912 (80.7)	
1	68 (15.3)	308 (13.0)	
2	17 (3.8)	93 (3.9)	
3	11 (2.5)	55 (2.3)	
Missing	167	1215	0.467
**Previous therapeutic abortions**			
0	429 (96.2)	2234 (94.0)	
1	17 (3.8)	143 (6.0)	
Missing	167	1206	0.065
**Previous caesarians**			
0	408 (91.9)	2192 (92.5)	
1	36 (8.1)	177 (7.5)	
Missing	169	1214	0.642
**Smoking history**			
Current smoker	44 (9.5)	278 (11.2)	
Former smoker	28 (6.1)	146 (5.9)	
Never smoked	274 (59.4)	1463 (59.0)	
Not known	115 (25.0)	592 (23.9)	
Missing	152	1104	0.751
**Smoking while pregnant**			
No	317 (68.8)	1667 (67.2)	
Yes	40 (8.7)	272 (11.0)	
Not known	104 (22.6)	540 (21.8)	
Missing	152	1104	0.338
**Alcohol referral to intervention nurse**			
No	604 (98.5)	3470 (96.9)	
Yes	9 (1.5)	113 (3.2)	
missing	0	0	0.022
**Units of alcohol drunk now**			
Nil	505 (94.4)	2860 (91.6)	
At least one unit	30 (5.6)	262 (8.4)	
missing	78	461	0.028
**Units drunk daily before pregnant**			
Nil	488 (90.5)	2694 (85.9)	
1–4	41 (7.6)	412 (13.1)	
5+	10 (1.9)	31 (1.0)	
missing	74	446	< 0.001
**Alcohol intervention required**			
No	446 (72.8)	2369 (66.1)	
Yes	167 (27.2)	1214 (33.9)	
missing	0	0	0.001
	**Median IQR**	**Median IQR**	
**Estimated gestation**	39 (38–40)	39 (38–40)	0.100
**Birthweight**	3366 (3050–3696)	3394 (3066–3725)	0.483
**CO Carbon Monoxide**	1 (1–2)	1 (1–2)	0.229

## Discussion

Passive consent to draw additional blood for research purposes at routine antenatal venepuncture resulted in a high proportion of women (85%) giving a sample. Whilst this study did not compare directly with consent for an alcohol study, it is likely that this high recruitment rate means that the prevalence of alcohol consumption in pregnancy in the group who had an extra sample taken is closer to that of the whole population than in studies where the nature of the study in relation to alcohol is disclosed. In other words, this method is likely to have reduced sampling bias compared to asking women to give blood for an alcohol study where those who drink alcohol often opt out.

Our pilot study [18] used anonymous testing of residual stored routine pregnancy blood samples similar to a recent study from the Netherlands [19]. Residual samples will have the least bias in this pregnant population who all require routine blood tests. We were not able to repeat anonymous residual sample testing as Carbohydrate Deficient Transferrin estimation requires a serum sample no longer routinely collected during pregnancy.

This paper compares demographic and other variables for pregnant women who had an extra blood sample taken for the ACDS at their antenatal booking appointment, under an unusual consent procedure, with those women who did not have a sample taken. As the blood samples will be used to assess the utility of a blood alcohol assay in predicting the risk of future child development problems, it is important for the study to understand any potential bias that this method of sampling may have introduced.

The number of women from whom the extra sample was collected was high at 85%, indicating that sampling had likely become routine practice. The reasons for the lack of sample in 15% of women are unclear. Some women may have opted out proactively, though there were no reports of this. Midwives may have sometimes forgotten to take the extra sample. Some midwives may have routinely asked women if they had seen the study ‘generic’ information sheet, and if women said that they had not, these midwives may not have taken the extra sample, although asking women if they had read the PIS was not a requirement of the passive consent process approved by the ethics committee. It is likely that multiple reasons apply, and the true reasons cannot be ascertained with any certainty, however the latter above was felt to be the most likely explanation by senior/research midwives who were supporting the study (and were fully informed).

The group not giving the sample was younger and less affluent than the majority from whom samples were collected. Older, better educated, more affluent women are more likely to report drinking in pregnancy in the UK in anonymized or retrospective surveys [20–22], however most such disclosures are of regular consumption of small quantities of alcohol. It is not known if this is a true difference in drinking or a reporting difference. Similarly, it is not currently possible to obtain reliable data on the demographics of heavier alcohol consumption, because of the lack of a ‘gold standard’ biomarker or reliable self-report test as discussed above. In this study, there was a significant difference between the two groups, with those who had an extra sample taken more likely to self-report alcohol use. This may indicate residual bias as women who drink but do not admit to alcohol use during pregnancy may be more likely to opt out of any research involvement. There was no evidence to suggest that the purpose of the study became known to midwives or pregnant women, so any bias in terms of alcohol consumption between the two groups is likely to be less than if fully informed consent had been employed.

The strengths of this cohort arise from the unusual passive consent procedure, with incomplete disclosure, which enabled a higher proportion of women to be included in the study than would otherwise have been the case, and reduced likely sampling bias due to non-participation of women who were drinking alcohol in pregnancy. The study had the full support of senior and research midwives in the NHS, who facilitated the training of frontline midwives and the routine collection of samples without breaching confidentiality about the intended testing of the ACDS samples for an alcohol biomarker. It is not possible to rule out any bias in the sample, however, as some women who are drinking heavily in pregnancy may have been more likely to opt out even of a generic study, given the level of anxiety around what is considered a stigmatized or sometimes even deviant behaviour.

### Discussion of ethics

The consent procedure used in this study was passive, and women did not know that the samples were intended for the ACDS. The issue of research without full informed consent has been extensively discussed in relation to three recent trials of online alcohol interventions in students [14, 23]. In these trials, the students did not know the true purpose of the research, and in two cases they did not know that they were participating in a trial. Subsequent commentaries on the ethics of these cases agreed on the necessity to very seriously consider the pros and cons of compromises to consent, but did not, however agree on the balance of harms in the studies in question [24–29]. The most ethically problematic aspect may be the practice of studying people without any consent [29]. Our study used passive consent, whereby women who read the PIS were informed that an extra sample would be taken for research purposes as above, and they had the opportunity to opt out on that basis. Some women may have had a sample taken without having read or fully understood the PIS. Passive consent was used to avoid burdening midwives, to reduce sampling bias, and to ensure a high enough sample size for the research to be viable. This is similar to routine practice in this setting where women are informed about routine antenatal testing that any residual blood leftover after testing is stored for anonymous use for research (subject to ethical and safe haven approval) unless they actively opt-out. The inclusion of a separate PIS may have increased the likelihood of women being aware of our research, compared to the leftover blood research information which is included on one page of a lengthy booklet. The value or risks of debriefing participants after the study is complete also need to be considered [23, 26–28] particularly given the potential impact on trust and participation in future research and researchers. We gave serious consideration to debriefing midwives or women, and discussed it further with senior midwifery colleagues, but on balance feel that to do so would cause harm in this case. We have also removed all identifying information from this paper about the study city, to minimize the risk of deductive disclosure.

Although widely accepted as the ethical foundation of human health research, the Helsinki Declaration (https://www.wma.net/policies-post/wma-declaration-of-helsinki-ethical-principles-for-medical-research-involving-human-subjects/) has also been criticized for taking an individualistic approach. The central issue is whether the pursuit of the greatest good for society as a whole should take account of wider interests beyond those pertaining to the research participants themselves [30]. On balance, we agree with Hendershot et al. as they argue that: ‘the delivery of many public health interventions involves deferring individual autonomy to promote population health, [and therefore] studying candidate interventions under similar conditions is justified—and possibly necessary for maximizing the public health yield of intervention research’ [25]. Not doing research to better understand the nature and extent of serious problems caused by alcohol, even if it involves ethical risks, should not be an ethically comfortable position [23].

## Conclusion

The use of a passive consent process without disclosure of the specific focus of the study resulted in a high level of participation in the ACDS which aims to assess the value of a maternal blood biomarker for alcohol in predicting childhood developmental disorders. Younger and less affluent women were less likely to give a blood sample for the study. Those who did not give a sample were less likely to self-report alcohol use or need intervention for that use. The group who had an extra sample collected are therefore likely to include most women who drank alcohol during pregnancy. Critically, we had no evidence that the true purpose of the study was disclosed to midwives or pregnant women. Given the stigma associated with alcohol consumption in pregnancy, it is highly likely that the resulting cohort is much less biased than if the true purpose of the study had been disclosed.

Whilst it requires careful ethical consideration and justification, this method may be of value in other studies of stigmatized behaviours. In this case, testing of the blood samples for an alcohol biomarker (CDT) and successful data linkage with future developmental outcomes will enable assessment of the predictive value of maternal CDT at booking appointment.

If residual routine blood drawn in pregnancy with the least sampling bias is not available, this research cohort design may be used to examine the utility of alcohol screening assays in early pregnancy when prompt intervention could improve outcomes for both mother and child.

## Data Availability

Materials and data are not available for others to use.

## Abbreviations

ACDS: Alcohol and Child Development Study
UK: United Kingdom
PIS: Patient Information Sheet
NHS: National Health Service
FASD: Fetal Alcohol Spectrum Disorders
HIV: Human Immunodeficiency Virus
CO: Carbon Monoxide
BMI: Body Mass Index
SIMD: Scottish Index of Multiple Deprivation
CDT: Carbohydrate Deficient Transferrin
SIRS: Scottish Immunisation & Recall System
